# High susceptibility of magpie (*Pica pica*) to experimental infection with lineage 1 and 2 West Nile virus

**DOI:** 10.1371/journal.pntd.0006394

**Published:** 2018-04-10

**Authors:** Nereida Jiménez de Oya, María-Cruz Camacho, Ana-Belén Blázquez, José-Francisco Lima-Barbero, Juan-Carlos Saiz, Ursula Höfle, Estela Escribano-Romero

**Affiliations:** 1 Departamento de Biotecnología. INIA, Madrid, Spain; 2 Grupo de Sanidad y Biotecnología SaBio, Instituto de Investigación en Recursos Cinegéticos IREC, CSIC-UCLM-JCCM, Ciudad Real, Spain; Centers for Disease Control and Prevention, UNITED STATES

## Abstract

West Nile virus (WNV), a zoonotic pathogen naturally transmitted by mosquitoes whose natural hosts are birds, has spread worldwide during the last few decades. Resident birds play an important role in flavivirus epidemiology, since they can serve as reservoirs and facilitate overwintering of the virus. Herein, we report the first experimental infection of magpie (*Pica pica*) with two strains of West Nile virus, lineages 1 (NY-99) and 2 (SRB Novi-Sad/12), which are currently circulating in Europe. Magpies were highly susceptible to WNV infection, with similar low survival rates (30% and 42.8%) for both lineages. All infected magpies developed viremia detectable at 3 days post-infection with titers above those necessary for successful transmission of WNV to a mosquito. Neutralizing antibodies were detected at all time points analyzed (from 7 to 17 days post-infection). WNV genome was detected in the brains and hearts of all magpies that succumbed to the infection, and, in some of the surviving birds. WNV-RNA was amplified from swabs (oral and cloacal) at 3, 6 and 7 days post-infection and feather pulps, from 3 to 17 days post-infection, of infected animals. Even more, infectious virus was recovered from swabs up to 7 days post-infection and from feather pulps up to 10 days post infection. Sham-infected control animals were negative for viremia, viral RNA, and antibodies. These results suggest that the magpie, which is one of the most abundant corvid species in Europe, could represent a source of WNV transmission for birds and humans. Our observations shed light on the pathogenesis, transmission, and ecology of WNV and can benefit the implementation of surveillance and control programs.

## Author summary

Birds play an important role in the epidemiology of flaviviruses such as West Nile virus (WNV) since birds are natural hosts and facilitate hibernation of the virus in periods of absence of mosquitoes that transmit the virus. Since it has been proposed that magpies play an important role in an endemic WNV cycle in human habitats in Europe, we conducted the first experimental infection of magpie with the two WNV lineages currently circulating in Europe. We observed high susceptibility of magpie to WNV infection with virus titers higher than those necessary for the successful transmission of WNV to a mosquito and often resulting in death. Likewise, we detected elevated titers of neutralizing antibodies in all the samples tested as well as the viral genome in the organs, oropharyngeal and cloacal swabs and feather pulps of the infected animals. Our results suggest that the magpie, which is one of the most abundant corvid species in Europe, could be a source of WNV transmission to other birds and humans, which expands the knowledge about WNV pathogenesis, transmission and ecology, that benefits monitoring and control programs.

## Introduction

West Nile virus (WNV) is an arbovirus (arthropod-borne virus) belonging to the family Flaviviridae, genus *Flavivirus*, which is maintained in an enzootic cycle involving several species of birds and mosquitoes appertaining mainly to the *Culex pipiens* complex [1]. Occasionally, it can infect humans and horses, which are considered dead end hosts due to the low viremic titers that they develop, which are insufficient to infect mosquitoes and maintain the transmission cycle [2]. While the majority of human WNV infections are sub-clinical, approximately 20% of patients suffer West Nile fever with flu-like symptoms and 1% develop a severe neuro-invasive potentially fatal disease [1].

After reaching the American continent in 1999, WNV spread around the globe, and is now considered the most widespread arthropod-borne virus in the world, and the main causative agent of arboviral encephalitis in the U.S. [3]. In Europe, only lineage 1 strains were circulating until isolation of a lineage 2 strain from a goshawk in Hungary in 2004 [4]. Since then, lineage 2 strains have been responsible for high bird mortality and dozens of human deaths in several Southeast European countries [5].

The pivotal role of migratory birds in spreading WNV infection is well documented [1]. Resident birds are also considered important in maintaining WNV circulation in nature [6]. In fact, WNV overwintering in Europe by local birds and mosquitoes has been suggested [4].

Pathogen transmission dynamics are related to host-feeding patterns of mosquitoes, resident or migratory bird behavior, and host susceptibility to the virus. For instance, the high mortality rates observed in WNV lineage 1 infected birds in Israel and North America, particularly among corvids [7], have not been observed in Europe, where lineage 1 viruses are circulating [8]. Nevertheless, WNV has been isolated from dead birds in Europe, including Passeriformes like magpie (*Pica pica*) [4, 6, 9–13].

During initial epidemics in the US, crows were considered to play an important role as virus amplifiers. It has been proposed that corvids, including magpie, are also involved in a WNV endemic cycle in human habitats in Europe [14]. Viral RNA has been detected in 5 to 9% of the tested population [12, 15] and neutralizing antibodies in 1.5 to 11% [16–18]. However, in order to consider the magpie as an infectious source of WNV transmission, it should develop a competent viremia and/or shed virus, aspects that have never been explored before. Therefore, herein we described the first experimental infection of magpie, one of the most abundant corvid species in Europe, in order to examine the pathogenesis of WNV infection in this host and elucidate the possible role of these birds in WNV ecology.

## Materials and methods

### Experimental design

Magpies were captured under permit 346760 of the regional government of the autonomic Community of Castilla-La Mancha using walk-in cage traps in several hunting locations in South-Central Spain. Traps were checked daily and, upon capture, birds were aged based on plumage and molt patterns, sampled, and tested for the presence of WNV-RNA by consensus flavivirus real time RT-PCR [19], and the presence of flavivirus antibodies by a commercial blocking ELISA (Ingenzim West Nile Virus, Ingenasa, Madrid, Spain), respectively. Birds that tested negative were transferred to mosquito-net covered flight cages at the experimental farm of the IREC (Instituto de Investigación en Recursos Cinegéticos, Ciudad Real, Spain). Captures took place between April and June 2017 and only individuals younger than a year old were selected. Magpies were maintained for a maximum of two months in groups of ten in mosquito-proof flight cages with *ad libitum* food and water supply, and were weighed and re-tested on a monthly basis to confirm well-being and lack of exposure to Flaviviruses. A final group of 34 magpies was transported to the biosafety level 3 (BSL-3) facilities at INIA (Instituto Nacional de Investigación y Tecnología Agraria y Alimentaria, Madrid, Spain), where they were housed in 3 separate cages (11–12 birds/cage). Each cage was equipped with a net-covered flight cage with several fixed and one free-swinging perch with artificial grass cover, rough cardboard lining on the ground, and enough space for free flight. After one week of adaptation, animals were weighed and blood was collected via the jugular vein for preinoculation serology (0 days post-inoculation, d.p.i.). Then, they were subcutaneously inoculated in the neck with 5x10^3^ plaque forming units (p.f.u.)/bird of WNV diluted in 100 μl of Eagle Minimum Essential Medium (EMEM, BioWhittaker, Lonza, Verviers, Belgium). One group of magpies (n = 12) was infected with NY-99 WNV lineage 1 strain (GenBank accession no. KC407666), another group (n = 11) with SRB Novi-Sad/12 WNV lineage 2 strain (GenBank accession no. KC407673), and the third negative control group (n = 11) was similarly sham-inoculated with medium. Two magpies per group were euthanized at 3 d.p.i. by intravenous injection of a sodium pentobarbital overdose.

All animals were handled in strict accordance with the guidelines of the European Community 86/609/CEE and the protocols were approved by the Committee on Ethics of animal experimentation of our Institution (INIA permit number 2017–01). Food and water were provided *ad libitum* throughout the experiment. Magpies were monitored daily for clinical signs, and those showing severe clinical signs were anesthetized and euthanized, as were all surviving animals at the end of the experiment (17 d.p.i.).

### Sampling

At 3, 7, 10 and 17 d.p.i., all surviving animals were weighed and sampled (blood, feathers, and oropharyngeal and cloacal swabs). Blood samples (0.5 ml per bird) were collected via the jugular vein and allowed to coagulate at 4°C overnight prior to centrifugation at 5,000 rpm for 10 minutes. Pulps of growing feathers and swabs were placed into 0.5 and 1 ml of EMEM medium, respectively. Any dead or euthanized magpie was subjected to full detailed necropsy and tissues were collected, splitted, and stored in PBS at -80°C and in 10% buffered neutral formalin for further virological and histopathological analyses, respectively. Additionally, swabs and feather pulps were also collected from dead and euthanized birds.

### Immunological and viral assays

WNV-specific neutralizing antibodies to both viral lineages (1 and 2) were detected in serum samples by plaque reduction neutralization test (PRNT) on Vero cells using twofold serial serum dilutions, as previously described [6]. Titers were calculated as the reciprocal of the serum dilution, diluted at least 1:20, which reduced plaque formation ≥ 90% (PRNT_90_) relative to samples incubated with negative control pooled sera.

Collected sera, feather pulps and oral and cloacal swabs were also tested for infectious WNV by plaque assay on Vero cell culture as previously reported [20].

### Detection of WNV-RNA in organs, swabs and feathers

Tissues (brains and hearts) were thawed and weighed prior to homogenization in a TissueLyser II mixer (2 minutes at 30 cycles/s; Qiagen, Germany) in 0.75 ml EMEM. Resulting homogenates were clarified by centrifugation (12,000 rpm for 5 minutes) and stored at -80°C. Viral RNA from the processed tissues, as well as from oropharyngeal and cloacal swabs and feather pulps, was extracted using a QIAcube extractor (Qiagen, Germany) according to the instructions provided by the manufacturer.

Lineage 1 viral RNA was detected by real-time RT-PCR as previously described [21] using a High Scriptools-Quantimix Easy Probes kit (Biotools). For lineage 2 WNV-RNA quantification primers used were forward, 5’-CAGACCACACTCTAGTG-3’, and reverse, 5’-CCCACGCGGCCATAA-3’; enclosing nucleotides 10691 to 10793 of the WNV, SRB-Novi Sad/12 strain [GenBank accession no. KC407673; [6]]. Viral RNA was quantified as genomic equivalents (GE) to p.f.u. by comparison with RNA extracted from previously titrated samples [20].

WNV-RNA was amplified from samples of surviving animals by conventional RT-PCR (SuperScript One Step RT-PCR system, Invitrogen, Carlsbad, CA, USA) as described [20] using specific primers (forward, 5’- CCTTGGAATGAGCAACAGAGACTT -3’, and reverse, 5’- GTGTCAATGCTTCCTTTGCCAAAT -3’; enclosing nucleotides 985 to 1320 of WNV NY99 strain, GenBank accession no. KC407666) and bidirectionally sequenced (Macrogen, The Netherlands).

### Statistical analyses

Statistical analyses were performed using Graph Pad Prism for Windows, version 6 (Graph Pad Software, Inc., San Diego, CA, 2005). Kaplan-Meier survival curves were analyzed by a log-rank test. Mean survival time (MST) was calculated for every group of inoculated magpies. Two-way analysis of variance (ANOVA) with Bonferroni’s correction for multiple comparisons was used to evaluate the weight differences of the animals along the experiment. Unpaired t-test was used to compare viremia between the groups infected with the two lineages used. Statistically significant differences are indicated by asterisks * (p<0.05), ** (p<0.01), *** (p<0.001).

## Results

**Fig 1:**
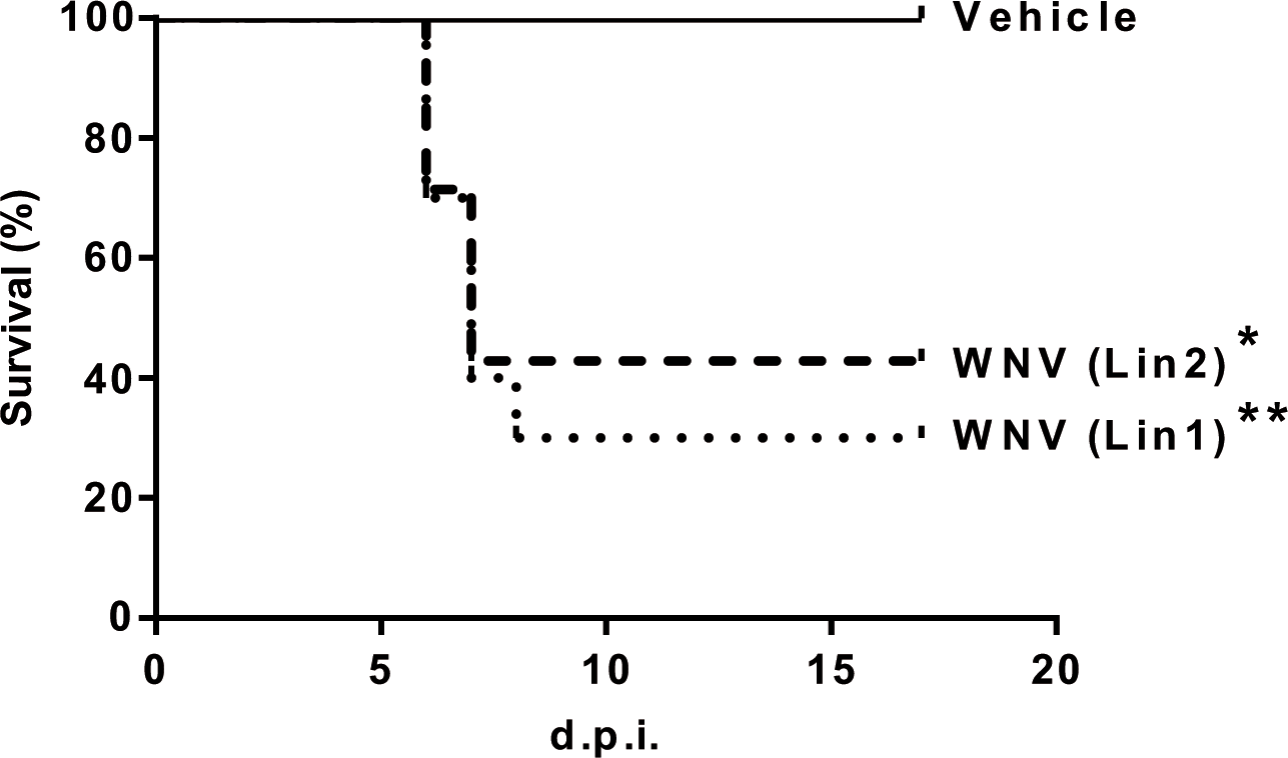
High mortality of magpies after WNV infection. Survival rates in birds infected with WNV-Lineage 1, WNV-Lineage 2 or sham-inoculated. Asterisks represent statistically significant differences between infected animals and controls (*P<0.*05*, **P<0.*01*).

**Fig 2:**
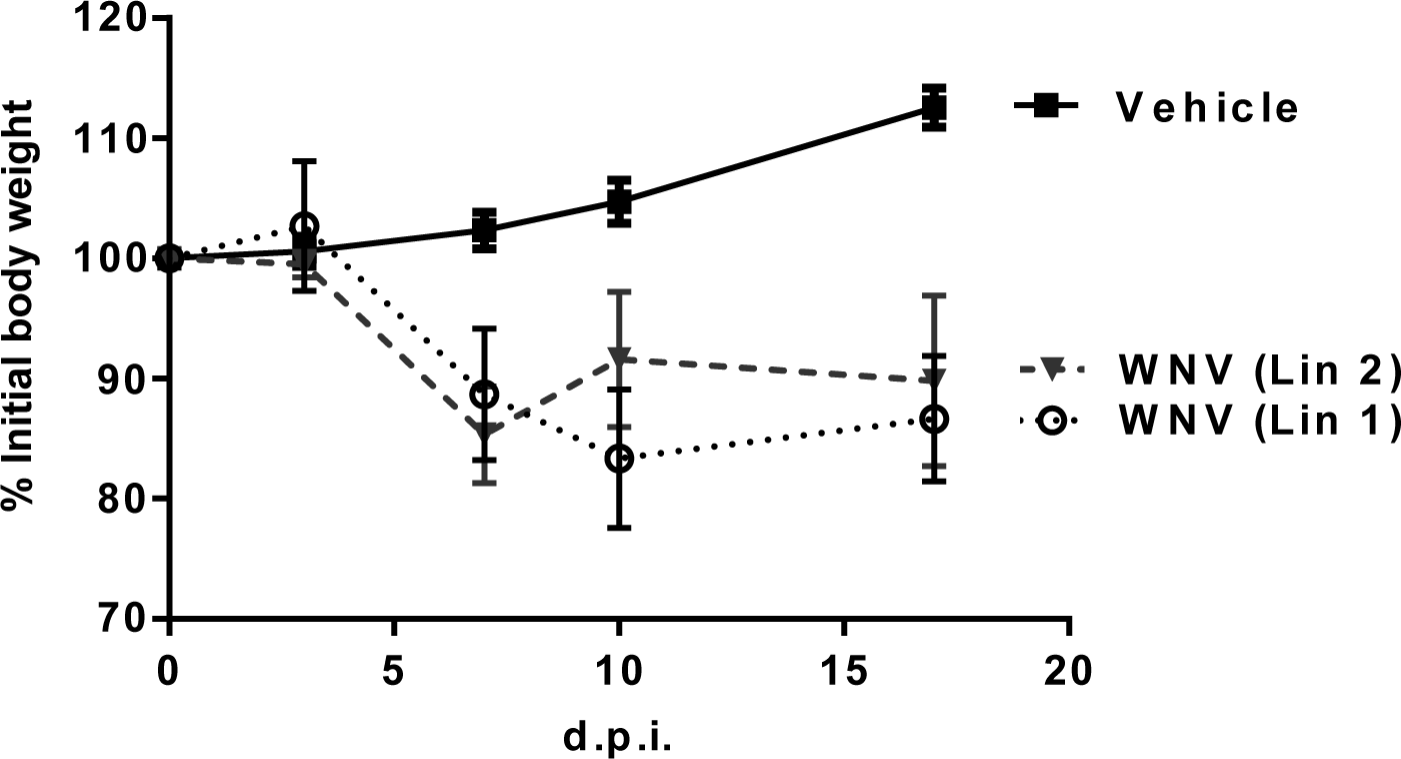
Infected magpies exhibited significant weight loss. Percentage of weight change during the experiment in WNV-Lineage 1, WNV-Lineage 2, and sham-inoculated birds. Statistically significant differences (*P<0*.*05*) with control magpies were recorded at days 7, 10 and 17 p.i.

A low survival rate was observed in magpies infected with WNV lineage 1 (30%) and 2 (42.8%), with mean survival times of 6.7 (range 6–8) and 6.5 (range 6–7) d.p.i., respectively (Fig 1). Clinical signs in magpies that died included lethargy, ataxia, inability to fly, and leg paralysis. Death typically occurred less than 10 hours after first showing clinical signs. Surviving, infected birds were lethargic 6–12 days post-infection. All infected magpies showed significant weight loss until they died. Surviving birds gained weight from 7 d.p.i. while control magpies did so throughout the course of the experiment (Fig 2).

**Fig 3:**
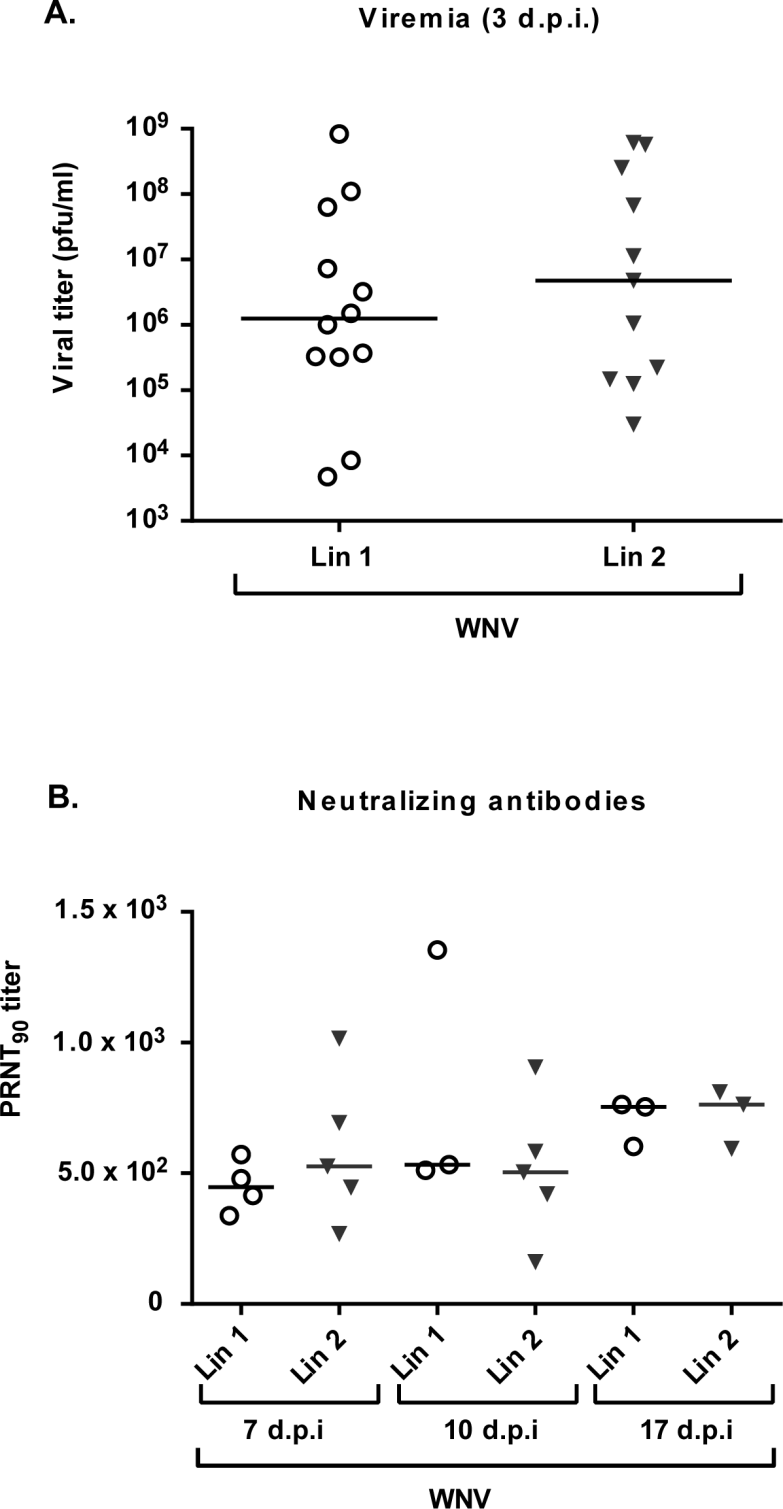
Infected magpies displayed high viremia and neutralizing antibody titers. (A) Viremia titers (p.f.u/ml) in birds infected with WNV-Lineage 1 and WNV-Lineage 2 at 3 d.p.i. (B) Neutralization titers (PRNT_90_) in birds infected as in (A) at days 7, 10 and 17 p.i.

Serum samples were tested for infective virus at different time points (3, 7, 10 and 17 d.p.i.) and positive samples were only detected at 3 d.p.i. in infected magpies, with high titers (range 5x10^3^-8x10^8^ p.f.u/ml) in both viral lineages (Fig 3). WNV-specific neutralizing antibodies were detected in all tested infected magpies from one week after infection until the end of the experiment, regardless of the infecting isolate (Fig 3B). PRNT_90_ titers ranged from 1.6x10^2^ to 1.35x10^3^, showing a discreet tendency to increase in surviving animals. All samples from sham-inoculated magpies resulted negative.

**Fig 4:**
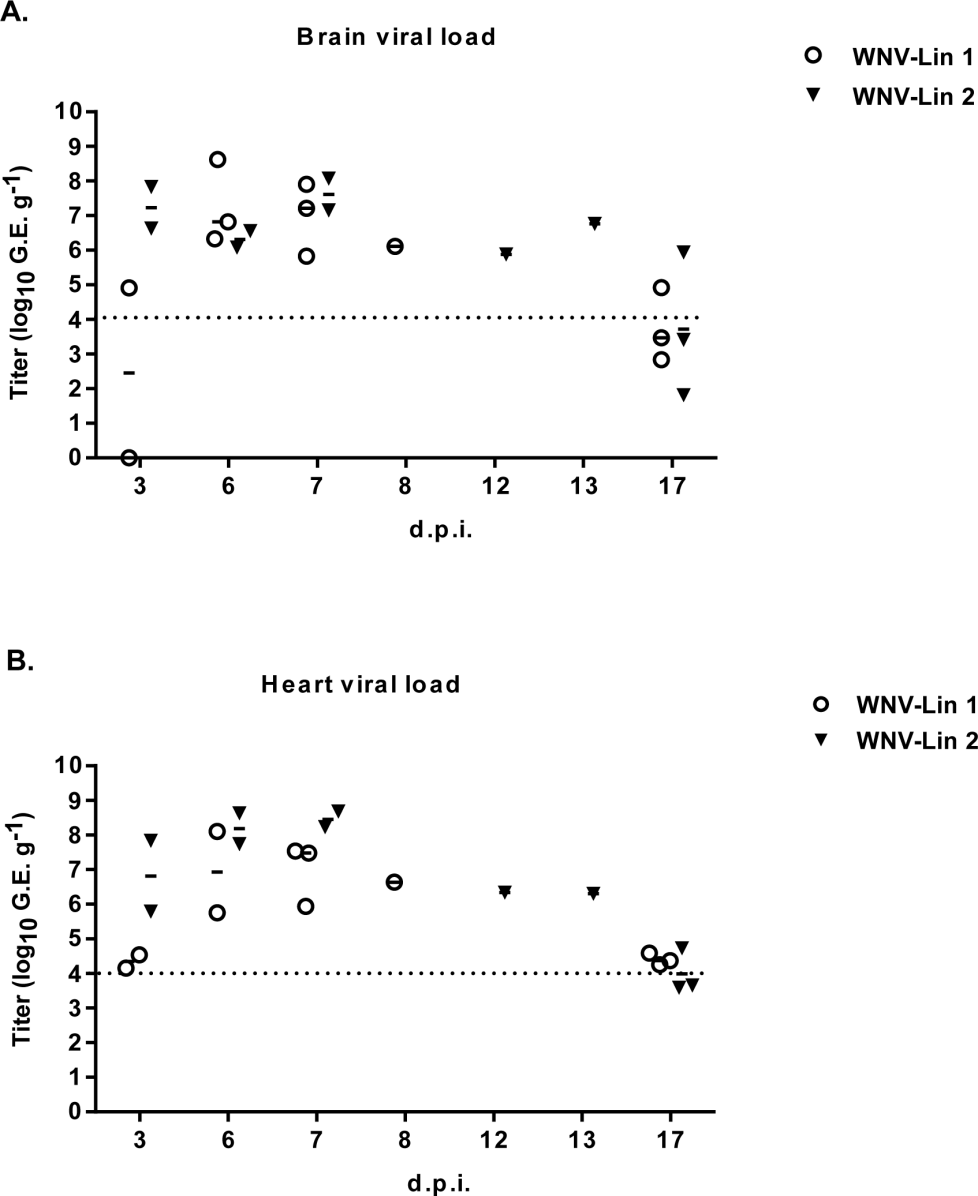
Detection of WNV-RNA in brains and hearts of infected birds. WNV-RNA titers (represented as genome equivalents per gram of organ) in brains (A) and hearts (B) of birds infected with WNV-Lineage 1 or WNV-Lineage 2 at different time post infection. Dashed line represents limit of detection of the assay.

**Fig 5:**
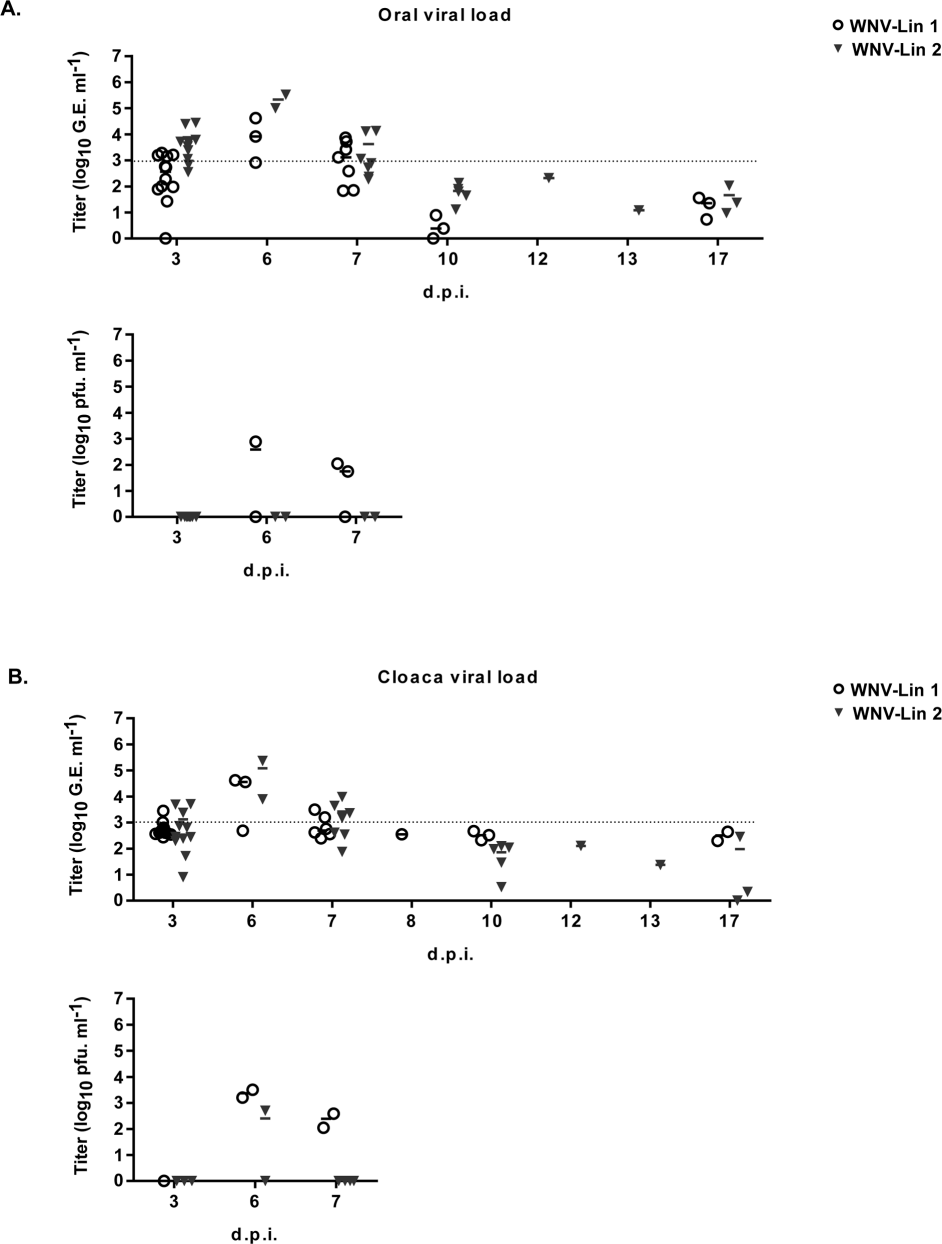
Detection of WNV-RNA and infectious virus in cloacal and oral swabs. WNV-RNA titers (represented as genome equivalents per milliliter, upper panels) and infectious virus (represented as pfu/ml, lower panels) in oral (A) and cloacal (B) swabs of birds infected with WNV-Lineage 1 or WNV-Lineage 2 at different time post infection. Dashed line represents limit of detection of the qRT-PCR assay.

**Fig 6:**
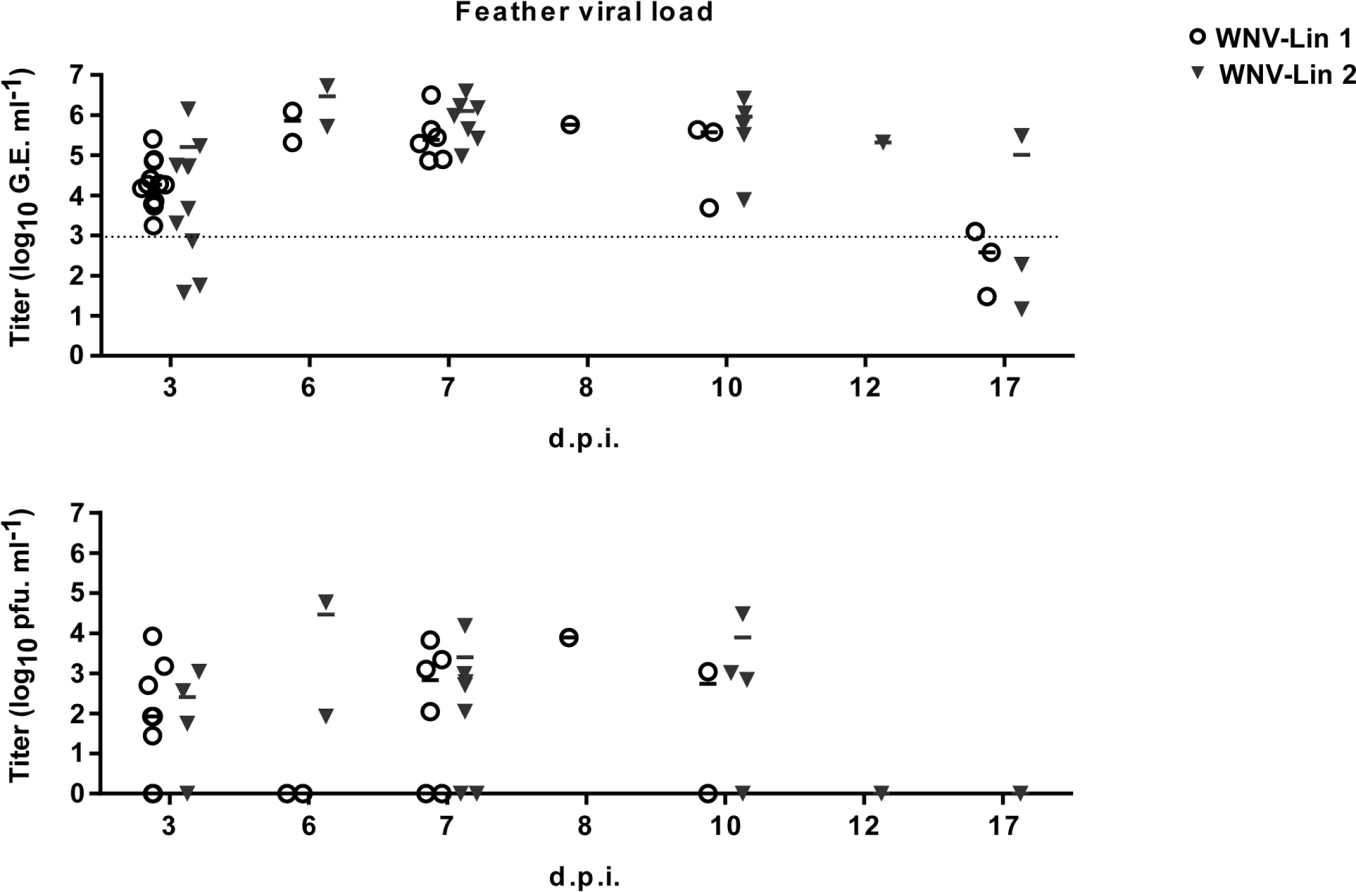
Detection of WNV-RNA and infectious virus in feather pulps. WNV-RNA titers (represented as genome equivalents per milliliter, upper panel) and infectious virus (represented as pfu/ml, lower panel) in feather pulps of birds infected with WNV-Lineage 1 or WNV-Lineage 2 at different time post infection. Dashed line represents limit of detection of the qRT-PCR assay.

Two magpies per group were euthanized at 3 d.p.i. to analyze the presence of WNV-RNA in their hearts and brains. Viral genome was detected in all samples from infected magpies, except in the brain of one lineage 1 infected bird (Fig 4). The initial organ sampling schedule was abrogated due to the high mortality observed, so only organs from animals that succumbed to the infection and from surviving birds at the end of the experiment were analyzed. All samples from dead birds were positive regardless of the infecting lineage. WNV-RNA was also detected in the brains from two surviving magpies, each one infected with a different lineage, as well as in the hearts of all surviving animals infected with lineage 1 virus, and in one of the three lineage 2 infected surviving birds (Fig 4). These results were confirmed by conventional RT-PCR and sequencing. WNV-RNA was also amplified from swabs (oral and cloacal) of infected birds from 3 to 7 d.p.i., peaking at 6 d.p.i. with no viral genomes detected in oropharyngeal or cloacal swabs after 7 days d.p.i (Fig 5A and 5B, upper panels). Presence of infectious virus was also analyzed in some representative WNV-RNA positive samples (Fig 5A and 5B, lower panels). On the other hand, almost all feather pulps from infected birds were WNV-RNA positive from 3 to 10 d.p.i., and in some of them virus could be also recovered (Fig 6). Notably, at 17 d.p.i., feather samples from one magpie of each group were also WNV-RNA positive, results that were confirmed after sequencing of RT-PCR amplicons. The two magpies that died at 12 and 13 d.p.i. had WNV-RNA in their brains, hearts, and feathers, but not in their swabs. Both individuals had a severe fungal pneumonia and airsacculitis, presumably caused by *Aspergillus* sp. that has to be considered the primary cause of death.

## Discussion

Magpie is one of the most abundant corvids in Europe [22] and it is suspected to be a source of WNV transmission. Using experimental infections, we showed that magpies are highly susceptible to fatal and nonfatal infections with WNV, elicit neutralizing antibodies, and develop viremic titers higher than those necessary for the successful transmission of WNV to mosquitoes [23]. Additional demonstration of infectious virus and viral genome in swabs and feather pulps, point to this species as a likely source of viral transmission.

WNV is maintained in nature in an enzootic cycle between its natural hosts -birds- and mosquitoes. In countries with continental climate, the enzootic cycle is apparently disrupted during the winter season, as vectors die or enter in diapause. Nevertheless, WNV vertical transmission in vectors has been described, indicating that the virus can overwinter in vertically infected mosquitoes [24], as well as in those horizontally infected that survive the increasingly warm and short winters.

Besides overwintering in mosquitoes, WNV can also be re-introduced by migratory birds. Moreover, climate change alters the migratory behavior of some wild bird species, some of which have now become residents in Europe, or shortened their migratory routes by wintering in Southern Europe instead of migrating to the African continent. In this new scenario, the idea of local birds acting as competent reservoirs that amplify and/or maintain the virus during winter in Europe is gaining force [14].

Magpie is widely distributed in Europe, representing the most abundant resident corvid, which is highly adapted to human environments. Additionally, magpie is one of the feeding preferences of *Culex pipiens*, a bridge WNV vector to humans [25]. However, only three isolations of WNV from magpies have been reported in Europe. In 2004, a WNV lineage 1 was isolated from a magpie in the Camargue, France [11], and in 2008 the virus was isolated from three magpies in Italy [12, 26]. Lineage 2 strain was detected in Greece in a hunter-harvested magpie in an area where human cases had occurred [27]. Despite briefing of hunters for recognition of encephalitis or collection of dead birds, no sick or dead magpies were recovered in the outbreak area. In contrast, WNV epidemics in America were associated with high bird mortality [28], leading to considerable declines in populations of related North American magpie species [28, 29]. A similar picture has been observed for American crows (*Corvus brachyrhynchos*) suffering substantial die-offs from WNV while no reports of mortality exist for the European carrion crows (*Corvus corone*), even though recent experiments have shown that both species are highly susceptible to WNV [30, 31].

We have performed the first experimental infection of magpies with 2 lineages of WNV isolates currently circulating in Europe. Birds were infected with the viral lineage 1 prototype NY-99, known to be highly pathogenic for the American magpie [29], and the SRB Novi-Sad/12 lineage 2 strain isolated from a dead Northern goshawk (*Accipeter gentilis*) during recent outbreaks in eastern Europe [6]. A high susceptibility to WNV infection was observed in magpies infected with both viral strains with survival rates of 30% and 42.8% for lineage 1 and 2, respectively. All infected magpies lost weight and elicited high neutralizing antibodies titers. In addition, all of them had high virus titers 3 d.p.i. (5x10^3^-8x10^8^ p.f.u/ml), demonstrating magpies can be a likely source of vector feeding transmission. In fact, host competence for WNV has been experimentally established based on viremia levels that range above 10^4^ to 10^5^ p.f.u/ml [30], and it should also be noted that the reduced mobility observed in viremic birds might increase exposure to host seeking mosquitos.

The high mortality rate recorded here is in line with previous data obtained from experimentally infected North American black-billed magpie (*Pica hudsonia*) [30]. In our experimental infection, the viral dose administered (5x10^3^ p.f.u/magpie) was in the range of the amounts inoculated by feeding mosquito [23]. Although lower pathogenicity with lower mortality from WNV in the European avian community has been proposed [8], our data suggest that the susceptibility of the European magpie to WNV could be underestimated. In fact, under the experimental conditions here reported, the development of signs prior to death took less than 24–48 hours.

The detection of infectious virus and viral RNA in feather pulps and cloacal and oral swabs suggests that magpie could also act as a source of horizontal transmission of WNV, not only within bird communities, but also to horses and humans, since they are highly adapted to human habitats and frequently forage on pastures. Even more, infections of birds after ingestion of infected animals has been described [30] and, therefore, death magpies can also be a source of WNV transmission for scavenger birds.

Two lineage 2 infected magpies died 12 and 13 d.p.i. (5 and 6 days later than any of the other infected animals). They succumbed presumably to aspergillosis, although WNV was also present in their brains, hearts and feathers. *Aspergillus fumigatus*, the primary cause of avian aspergillosis is an ubiquitous opportunistic pathogen and the combination of high concentrations of spores in the environment and impaired immunity of the bird are considered factors leading to the development of clinical disease [32]. Confinement, handling for sampling collection, and concomitant West Nile virus infection may more probably have contributed to development of the disease in these two individuals.

In summary, magpie is highly susceptible to West Nile virus infection; it amplifies the virus to sufficient levels to transmit it to other hosts and sheds it in considerable amounts, which probably contributes to maintain the viral life cycle. Since magpie lives close to human population, it should be a priority target in surveillance programs.
